# Evaluation of antimicrobial stewardship activities using antibiotic spectrum coverage

**DOI:** 10.1038/s41598-024-64944-2

**Published:** 2024-06-18

**Authors:** Michiya Tanuma, Takayuki Sakurai, Hidemasa Nakaminami, Masayo Tanaka

**Affiliations:** 1 Department of Pharmacy, NTT Medical Center Tokyo, 5-9-22 Higashi-gotanda, Shinagawa-ku, Tokyo, 141-8625 Japan; 2 Department of Clinical Microbiology, School of Pharmacy, Tokyo University of Pharmacy and Life Sciences, 1432-1 Horinouchi, Hachioji, Tokyo, 192-0392 Japan; 3 Department of Infectious Diseases, NTT Medical Center Tokyo, 5-9-22 Higashi-gotanda, Shinagawa-ku, Tokyo, 141-8625 Japan

**Keywords:** Antimicrobial agents, Antimicrobial stewardship, Antibiotic spectrum coverage, Antimicrobial use density, Days of antibiotic spectrum coverage, Days of therapy, Antimicrobials, Clinical microbiology

## Abstract

Recently, the days of antibiotic spectrum coverage (DASC) using the antibiotic spectrum coverage (ASC) score was reported as a new tool for measuring antimicrobial use. The days of therapy (DOT) are required to calculate the DASC, making it impossible to use when patient-level information is unavailable. Therefore, we have defined a new measure of antimicrobial use for antimicrobial spectrum coverage (AUSC) using antimicrobial use density (AUD) and ASC scores. In this study, we have investigated the use of antimicrobial agents retrospectively examined for monthly prescriptions between 2016 and 2022, and whether the AUSC could be used as a new measure. Our data showed that the AUD, AUSC, DOT, and DASC increased, whereas AUSC/AUD and DASC/DOT decreased over the study period. In addition, no correlation was found between DOT and DASC/DOT (ρ = − 0.093, *p* = 0.399), whereas there was a weak correlation between AUD and AUSC/AUD (ρ = − 0.295, *p* = 0.006). Therefore, in this study, the use of AUSC is considered less beneficial when DASC can be calculated based on DOT. On the other hand, in institutional settings where DOT cannot be calculated, AUSC may be useful as a new measure to evaluate antimicrobial use.

## Introduction

The Action Plan on Antimicrobial Resistance (AMR) was launched to promote the appropriate use of antimicrobial agents, and specific performance indicators for antimicrobial use have been established. Antimicrobial stewardship (AS) is a widely used approach to ensure the appropriate use of antimicrobial agents^1–3^. The de-escalation strategy for antimicrobial agents is an AS strategy for reducing the unnecessary use of broad-spectrum antimicrobial agents, which is expected to reduce the emergence of antimicrobial-resistant bacteria and medical costs. Antimicrobial use monitoring can be used as an indicator to evaluate AS activity and is an important source of information for promoting the appropriate use of antimicrobial agents. The World Health Organization's (WHO) Anatomical Therapeutic Chemical (ATC) and Defined Daily Dose (DDD) systems have been used to evaluate antimicrobial use^4^. Antimicrobial use density (AUD; DDDs/100 patients-days) and days of therapy (DOT) are commonly used to determine antimicrobial use in hospitalized patients^5^. The guidelines of the Infectious Diseases Society of America (IDSA) suggest using DOT over DDD wherever possible^5^. To calculate DOT, it is essential to have information on the number of days of medication at the patient level^6–8^. DOT calculations are possible when electronic medical records and antimicrobial aggregation systems are available; however, DOT calculations are difficult when systems have not been implemented. On the other hand, calculating the AUD does not require patient-level information, and aggregation is relatively easy. According to a survey, the adoption rate of electronic medical records is low in Japan^9^. Therefore, these indicators can be used by many medical institutions to evaluate antimicrobial stewardship in a simple and detailed manner. In addition, the AUD and DOT have several issues and do not reflect the antimicrobial susceptibility of bacteria or the spectrum of antimicrobial agents. Therefore, using the AUD and DOT as tools for evaluating AS should be interpreted with caution.

Recently, the days of antibiotic spectrum coverage (DASC), which uses the antibiotic spectrum coverage (ASC) score, has been proposed as a new tool for evaluating antimicrobial use^10,11^. The DASC is calculated from the DOT and ASC scores, which combine consumption and spectrum. A few reports of AS were assessed using the DASC in Japan, and the evidence is limited because it is a new indicator. It is difficult to calculate DASC in facilities where patient-level information is difficult to collect. Unlike DOT, AUD can be calculated using the total amount of antimicrobial agents used, making it possible for many hospitals to calculate AUD. The AMR Action Plan must be carried out on a regional basis. Therefore, indicators that clinics and medical institutions can easily calculate without electronic medical records or antimicrobial aggregation systems are needed. Therefore, we defined a new measure calculated from the AUD and ASC scores: antimicrobial use for antimicrobial spectrum coverage (AUSC). AUSC is a new measure that reflects antimicrobial usage and spectrum coverage. In addition, the AUSC provides a more detailed measure of antimicrobial agent use than the AUD. AUSC/AUD represents each hospital's average spectrum of antimicrobial agents. In this study, we calculated the AUSC and investigated whether it could be used as a new measure to assess AS activity in our hospital, together with the AUD, DOT, and DASC**.**

## Methods

### Patients studied and study period

This retrospective study was conducted at the NTT Medical Center in Tokyo, Japan, an acute care hospital with 594 inpatient beds. Antimicrobial use was retrospectively examined for monthly prescriptions between January 2016 and December 2022. This study was approved by the Ethics Committee of NTT Medical Center Tokyo prior to the study (approval number: 23-91). Owing to the retrospective and non-invasive nature of the study, the requirement for individual informed consent was waived. Disclosures about the study and an opt-out option were available for the patients on the hospital website. The study was conducted with full consideration of protecting patients’ personal information according to the privacy policy guidelines.

### Antimicrobial stewardship

During the observation period, the AS was led by one board-certified infection control pharmacy specialist (0.5 full-time equivalents [FTE]), one to two infectious disease physicians (total, 0.8 FTE), one clinical laboratory technician (0.5 FTE), and one to two nurses (total, 0.3 FTE). The total FTE for AS members was 2.0 FTE. AS monitored all inpatients for antimicrobial use and provided feedback every weekday. The number of interventions for the prescription of antimicrobial agents by the AS had a mean of 736.2 ± 301 (standard deviation) interventions per month. The number of recommendations for antimicrobial selection by AS had a mean of 67.0 ± 30.7 interventions per month; for de-escalation, 21.6 ± 15.6; for dose changes, 10.3 ± 8.4; and for stopping antimicrobial therapy, 11.8 ± 8.5. The acceptance rate of recommendations during the reporting period was 95.5%.

### Data collection

The AMR surveillance system is the Japan Surveillance for Infection Prevention and Healthcare Epidemiology (J-SIPHE)^12^. The AUD and DOT were calculated using the J-SIPHE application from the EF combined file, which consisted of the E file (medical treatment details) and F file (medical action details) used for medical billing in the Diagnosis Procedure Combination practice. The AUD and DOT can be calculated by submitting the EF files and using data files in the J-SIPHE application.

Additionally, the AUSC and DASC were calculated using the ASC scores defined in previous studies^10,13^. Furthermore, we calculated the AUSC/AUD and DASC/DOT, representing the average antibiotic spectrum provided to hospital inpatients. Patients treated with antiviral or antifungal agents for which ASC scores are yet to be determined were excluded. The definitions of the AUD, DOT, DASC, and AUSC are as follows:

Antimicrobial use density: (AUD)

 = [(total usage/defined daily dose) / (total number of patient days)] × 100.

Days of therapy: (DOT)

 = [total days used/total number of patient days] × 100.

Antimicrobial use for antimicrobial spectrum coverage: AUSC

 = antimicrobial use density × antibiotic spectrum coverage score.

Days of antibiotic spectrum coverage: DASC

 = days of therapy × antibiotic spectrum coverage score.

### Statistical analysis

We statistically analyzed trends in AUD, AUSC, AUSC/AUD, DOT, DASC, and DASC/DOT using linear regression. The regression coefficient β, 95% confidence interval (CI), and coefficient of determination R^2^ were used to calculate whether there was an increase or decrease. In addition, Spearman's correlation analysis was used to calculate the coefficient (ρ) of correlations between AUD, AUSC, and AUSC/AUD and between DOT, DASC, and DASC/DOT. SPSS version 24 (SPSS, Chicago, IL, USA) was used for all of the analyses.

## Results

**Figure 1:**
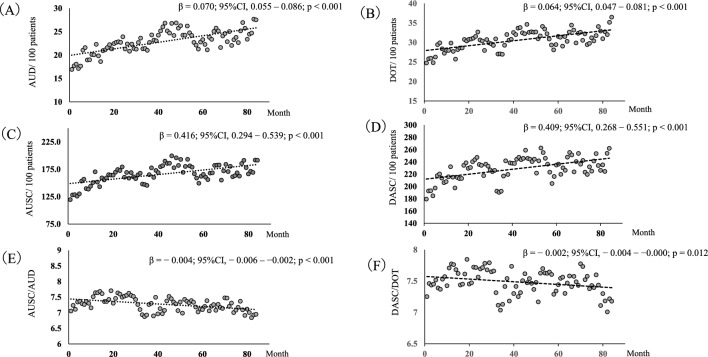
Monthly AUD/100 patients, AUSC/100 patients, AUSC/AUD, DOT/100 patients, DASC/100 patients and DASC/DOT trends at NTT Medical Center Tokyo during 2016–2022. AUD; antimicrobial use density, AUSC; antimicrobial use of spectrum coverage, DOT; days of therapy, DASC; days of antibiotic spectrum coverage.

AUD increased by 28.9% in 2022 when compared to that in 2016 (β = 0.070; 95% confidence interval [CI], 0.055–0.086; *p* < 0.001) (Fig. 1A). Carbapenems decreased by 20.0%, from 3.88 AUD to 3.11 AUD (*p* = 0.017); fluoroquinolones decreased by 2.0%, from 0.45 AUD to 0.44 AUD (*p* = 0.428), and aminoglycosides decreased by 91.0%, from 0.41 AUD to 0.04 AUD (*p* = 0.002). In contrast, penicillin increased by 94.1%, from 2.59 AUD to 5.03 AUD (*p* = 0.002); first-generation cephalosporins increased by 49.1%, from 3.28 AUD to 4.89 AUD (*p* < 0.001); third-generation cephalosporins increased by 13.6%, from 3.86 AUD to 4.39 AUD (*p* = 0.341); and fourth generation cephalosporins increased by 46.7%, from 0.82 AUD to 1.21 AUD (*p* = 0.224) (Table1). DOT increased by 18.0% in 2022 when compared to that in 2016 (β = 0.064; 95% CI 0.047–0.081; *p* < 0.001) (Fig. 1B). Similar to AUD, the DOT of carbapenems, fluoroquinolones, and aminoglycosides decreased, whereas that of penicillin, first-, third-, and fourth-generation cephalosporins, oxacephems, and cephamycins increased (Table 1). In addition, AUD/DOT was significantly increased by 10.0%, from 0.70 to 0.77 (β = 0.001; 95% CI 0.001–0.001; *p* < 0.001) during the study period.

**Table 1:** Comparing AUD and DOT trends at NTT Medical Center Tokyo between 2016 and 2022.

	AUD						DOT					
	β	95%CI			*p* value	*R*^*2*^	β	95%CI			*p* value	*R*^*2*^
Penicillins	0.412	0.233	–	0.590	0.002	0.876	0.000	− 0.001	–	0.000	0.053	0.560
1st-generation cephalosporins	0.308	0.216	–	0.401	< 0.001	0.936	0.009	− 0.027	–	0.045	0.546	0.077
2nd-generation cephalosporins	− 0.014	− 0.024	–	− 0.004	0.017	0.713	− 0.035	− 0.058	–	− 0.012	0.011	0.755
3rd-generation cephalosporins	0.129	− 0.186	–	0.443	0.341	0.181	0.000	− 0.122	–	0.123	0.993	0.000
4th-generation cephalosporins	0.054	− 0.046	–	0.153	0.224	0.277	0.048	− 0.073	–	0.170	0.354	0.173
Oxacephems / Cephamycins	0.022	− 0.110	–	0.153	0.688	0.035	0.025	− 0.215	–	0.266	0.798	0.011
Fluoroquinolones	− 0.022	− 0.988	–	0.044	0.428	0.129	− 0.002	− 0.048	–	− 0.043	0.895	0.004
Tetracyclines	0.011	− 0.015	–	0.037	0.320	0.196	0.006	− 0.022	–	0.035	0.580	0.065
Carbapenems	− 0.208	− 0.361	–	− 0.054	0.017	0.708	− 0.255	− 0.465	–	− 0.045	0.026	0.660
Aminoglycosides	− 0.056	− 0.080	–	− 0.031	0.002	0.874	− 0.146	− 0.197	–	− 0.092	< 0.001	0.910
Glycopeptides	0.167	0.074	–	0.260	0.006	0.810	0.201	0.063	–	0.338	0.013	0.738

*AUD* antimicrobial use density, *DOT* days of therapy, *β* regression coefficient, *R*^*2*^ determination coefficient, *CI* confidence interval.

The AUSC increased by 24.6% in 2022 when compared to that in 2016 (β = 0.416; 95% CI 0.294–0.539; *p* < 0.001) (Fig. 1C), and the DASC increased by 15.2% (β = 0.409; 95% CI 0.268–0.551; *p* < 0.001) (Fig. 1D). However, the AUSC/AUD reduced by 3.3% during the study period (β = − 0.004; 95% CI − 0.006 to − 0.002; *p* < 0.001) (Fig. 1E), and the DASC/DOT was reduced by 2.3% (β = − 0.002; 95% CI − 0.004 to − 0.000; *p* = 0.012) (Fig. 1F).

**Figure 2:**
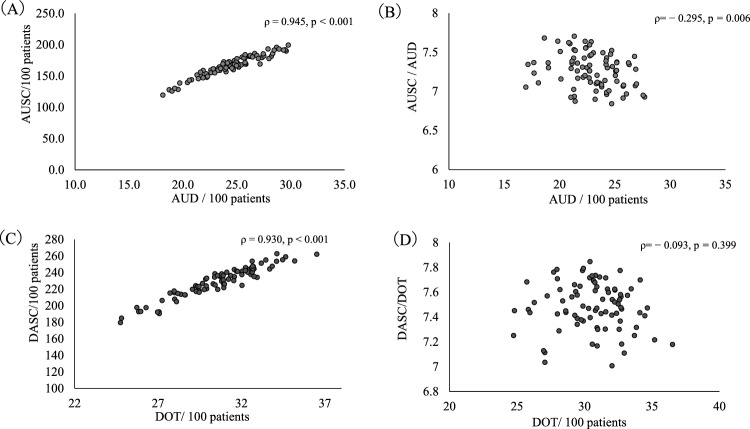
The scatterplot among AUD, AUSC, AUSC/AUD, DOT, DASC, and DASC/DOT. AUD; antimicrobial use density, AUSC; antimicrobial use of spectrum coverage, DOT; days of therapy, DASC; days of antibiotic spectrum coverage.

Examination of the correlations between AUD, AUSC, and AUSC/AUD revealed a strong correlation between AUD and AUSC (ρ = 0.945, *p* < 0.001) but a weak correlation between AUD and AUSC/AUD (ρ = − 0.295, *p* = 0.006) (Fig. 2A,B). Furthermore, correlations between DOT, DASC, and DASC/DOT revealed a strong correlation between DOT and DASC (ρ = 0.930, *p* < 0.001) but no correlations were found between DOT and DASC/DOT (ρ = − 0.093, *p* = 0.399) (Fig. 2C,D).

## Discussion

AUD and DOT do not reflect the spectrum of antimicrobial agents. In contrast, AUSC and DASC, including the ASC score, are composite metrics that simultaneously measure antimicrobial consumption and spectrum. AUSC is a new definition calculated from the AUD and ASC scores. This study examined its potential as a new definition following AUD, DOT, and DASC. This study suggests that AUSC may be used as an alternative to DASC as a spectrum indicator of antimicrobials in situations where DOT cannot be calculated. However, it should be noted that AUSC is affected by DDD and may not directly reflect the ASC score compared to DASC.

In this study, the total AUD and DOT increased during the study period. Carbapenems, fluoroquinolones, and aminoglycosides decreased AUD and DOT. In contrast, the AUD and DOT of penicillin and first-, third-, and fourth-generation cephalosporins, oxacephems, and cephamycins tended to increase. This suggests a shift towards more narrow-spectrum antimicrobial agents. This was indicated by the fact that AUSC/AUD and DASC/DOT decreased over this period.

This study evaluated a new definition of AUSC. The AUSC is an indicator that reflects the AUD and antimicrobial spectrum and is relatively easy to calculate, even in situations where the DOT cannot be calculated. During the study period, there was a significant increase in both AUSC and DASC. The potential reduced impact of the ASC score compared to the DASC should be considered when interpreting the AUSC. When considering antimicrobials with the same ASC score, one administered at a dose below the DDD would receive a lower AUSC score compared to another administered at a standard dose. Therefore, incorporating the concept of DDD into spectrum coverage indicators could make it impossible to accurately calculate spectrum coverage. In contrast, DASC is considered a more appropriate spectrum coverage indicator than AUSC because the ASC score is directly reflected in the spectrum coverage index. In Japan, the recommended dosage of some antimicrobial agents is set lower than the DDD. In this study, AUD/DOT was less than 1 (monthly mean 0.75 ± 0.03) and the daily dose was less than DDD. The discrepancy between the recommended dosage and the DDD may be the reason why the benefit of AUSC could not be derived.

Furthermore, significant decreases in AUSC/AUD and DASC/DOT may be due to a decrease in the unnecessary use of broad-spectrum antimicrobial agents in empirical therapy and a shift to narrow-spectrum antimicrobial agents, consistent with the AUD and DOT results. This suggests that AUSC/AUD and DASC/DOT can be used as indicators of de-escalation. When the DOT and DASC cannot be calculated, combining AUD as a usage indicator and AUSC/AUD as a spectrum indicator may be a more appropriate way to evaluate AS efforts.

On the other hand, DASC/DOT did not correlate with DOT. This study also suggests that the DASC/DOT can be employed as an independent indicator for evaluating the spectrum of antimicrobial agents used, as evidenced by previous studies^11^. However, a weak correlation was observed between AUSC/AUD and AUD, suggesting that AUSC/AUD was influenced by variations in DDD.

This study had several limitations. First, this was a single-center study in Tokyo, Japan, and the feasibility of these findings as indicators for evaluating antimicrobial agents in other centers needs to be investigated. Second, this study could not examine differences in patient backgrounds, such as disease type and severity, during the study period. Cefazolin is recommended for treating *Staphylococcus aureus* (*S. aureus*) bacteremia for 14 days for uncomplicated infections and 4–8 weeks for complicated infections, including infective endocarditis^14–18^. Combined and long-term antimicrobial therapies are recommended to treat infective endocarditis and other complicated infections. Consequently, AUDs and DOTs are high even with appropriate antimicrobial therapy, and it is unclear how the patients’ backgrounds affect the changes in AUD and DOT. Third, the spectrum of antimicrobial agents available for treating antimicrobial-resistant bacteria, such as extended-spectrum β-lactamase-producing bacteria and methicillin-resistant *S. aureus*, is broader than that for treating bacterial infections caused by antimicrobial-susceptible strains^19^. However, the present study did not examine the relationship between the number of resistant bacteria and AUSC or DASC. Finally, this study did not consider the COVID-19 pandemic. This may have had an impact on the diversity, severity, and pattern of antimicrobial prescriptions of hospitalized patients in this study.

## Conclusions

In this study, the use of the AUSC as a new measure was not found to be more useful than the DASC. The AUSC may not be an accurate definition in countries where antimicrobial doses are set below the DDD. Accordingly, this study suggests that the AUSC does not add much to the DASC, where the DASC can be calculated based on the DOT. However, when DOT cannot be calculated, trends in AUD and AUSC within hospitals can be useful indicators for assessing antimicrobial use. It is necessary to conduct multi-center database studies to verify the usefulness of AUSC.

## Data Availability

The datasets generated and/or analyzed during the current study are available from the corresponding author upon reasonable request.
